# The histone methyltransferase G9a as a therapeutic target to override gemcitabine resistance in pancreatic cancer

**DOI:** 10.18632/oncotarget.11256

**Published:** 2016-08-12

**Authors:** Mei-Ren Pan, Ming-Chuan Hsu, Chi-Wen Luo, Li-Tzong Chen, Yan-Shen Shan, Wen-Chun Hung

**Affiliations:** ^1^ Graduate Institute of Clinical Medicine, College of Medicine, Kaohsiung Medical University, Kaohsiung 807, Taiwan; ^2^ Research Center for Environmental Medicine, Kaohsiung Medical University, Kaohsiung 807, Taiwan; ^3^ National Institute of Cancer Research, National Health Research Institutes, Tainan 704, Taiwan; ^4^ Department of Pathology, Kaohsiung Medical University Hospital, Kaohsiung Medical University, Kaohsiung 807, Taiwan; ^5^ Division of Hematology/Oncology, Department of Internal Medicine, National Cheng Kung University Hospital, Tainan 704, Taiwan; ^6^ Institute of Clinical Medicine, College of Medicine, National Cheng Kung University, Tainan 704, Taiwan; ^7^ Department of Surgery, National Cheng Kung University Hospital, Tainan 704, Taiwan; ^8^ Institute of Basic Medical Sciences, National Cheng Kung University, Tainan 704, Taiwan

**Keywords:** lysine demethylase, drug resistance, interleukin-8, pancreatic stellate cell

## Abstract

Gemcitabine (GEM) resistance is a critical issue for pancreatic cancer treatment. The involvement of epigenetic modification in GEM resistance is still unclear. We established a GEM-resistant subline PANC-1-R from the parental PANC-1 pancreatic cancer cells and found the elevation of various chromatin-modifying enzymes including G9a in GEM-resistant cells. Ectopic expression of G9a in PANC-1 cells increased GEM resistance while inactivation of G9a in PANC-1-R cells reduced it. Challenge of PANC-1 cells with GEM increased the expression of stemness markers including CD133, nestin and Lgr5 and promoted sphere forming activity suggesting chemotherapy enriched cancer cells with stem-like properties. Inhibition of G9a in PANC-1-R cells reduced stemness and invasiveness and sensitized the cells to GEM. We revealed interleukin-8 (IL-8) is a downstream effector of G9a to increase GEM resistance. G9a-overexpressing PANC-1-R cells exhibited autocrine IL-8/CXCR1/2 stimulation to increase GEM resistance which could be decreased by anti-IL-8 antibody and G9a inhibitor. IL-8 released by cancer cells also activated pancreatic stellate cell (PSC) to increase GEM resistance. In orthotopic animal model, GEM could not suppress tumor growth of PANC-1-R cells and eventually promoted tumor metastasis. Combination with G9a inhibitor and GEM reduced tumor growth, metastasis, IL-8 expression and PSC activation in animals. Finally, we showed that overexpression of G9a correlated with poor survival and early recurrence in pancreatic cancer patients. Collectively, our results suggest G9a is a therapeutic target to override GEM resistance in the treatment of pancreatic cancer.

## INTRODUCTION

Pancreatic cancer is one of the most lethal malignancies in the world. The features of late diagnosis, highly metastatic ability and ineffective chemo- and radio-therapies lead to high mortality with a 5-year overall survival (OS) rate less than 5% [1]. Only 20–30% of pancreatic cancer patients could receive surgical resection at diagnosis, and 70–80% of patients were identified as unresectable, including locally advanced or metastatic disease [2].

Gemcitabine (GEM, 2′,2′-difluorodeoxycytidine) is a nucleotide analogue widely used in cancer treatment [3]. At present, GEM is an important chemotherapeutic drug for pancreatic cancer patients [4, 5]. However, the response rate is low and the improvement of 5-year OS is unsatisfied [6]. The reasons for a low response rate could attribute to the intrinsic chemoresistance to GEM or the acquired resistance developed after repeated exposure. Several mechanisms have been suggested to enhance GEM resistance. Nucleoside analogs like GEM are imported into cancer cells via specialized transporter systems including human equilibrative nucleosides transporters (hENTs) and concentrative nucleosides transporters and down-regulation of the transporters may increase drug resistance [7, 8]. Low expression of hENT1 in pancreatic cancer patients displays poor OS and disease-free survival (DFS) [9]. After entering cells, GEM needs to be phosphorylated by deoxycytidine kinase for activation [10]. Deficiency of deoxycytidine kinase is frequently found in pancreatic cancer cells and therefore contributes to inherent or induced GEM resistance in a portion of patients [11]. The cytotoxicity of GEM is mediated by its metabolite 2′,2′-difluoro dCDP (dFdCDP) and dFdCTP to inhibit ribonucleotide reductase [12]. Up-regulation of ribonucleotide reductase expression attenuates GEM cytotoxicity and provides another mechanism of GEM resistance [13].

Epigenetic regulations including DNA methylation and histone modification have also been shown to involve in GEM resistance. A global study of the DNA methylation patterns in pancreatic cancer genome showed that 289 CpG sites were differentially methylated in normal pancreas and pancreatic cancer [14]. Among the candidate genes identified, 23 were hypermethylated and 35 were hypomethylated in tumor parts. A metabolic enzyme glutathione-S-transferase μ 1 (GSTM1) and a transcription factor ONECUT2_E96_F were demonstrated to be useful for the prediction of GEM sensitivity. Expression of STAT1, an important transcription factor activated by the interferon signaling pathway, was shown to play a key role in the cellular response to GEM treatment [15]. Overexpression of 14-3-3σ has also been implicated in the acquired GEM resistance in pancreatic and breast cancer cells [16].

Study of three histone markers suggested that cellular histone modification patterns may predict prognosis and treatment response in resectable pancreatic cancer [17]. Up-regulation of histone deacetylase 3 (HDAC3) promoted GEM resistance in pancreatic cancer cells via modulation of cell cycle- and apoptosis-related genes and two HDAC inhibitors trichostatin A and 4-phenylbutyrate inhibited the proliferation of pancreatic cancer cells and enhanced the sensitivity to GEM [18–20]. By using PCR-based array approach, we showed that several histone modifying enzymes are up-regulated in GEM-resistant pancreatic cancer cells. We demonstrated that G9a (also known as euchromatic histone-lysine N-methyltransferase 2, EHMT2) plays an important role in the development of GEM resistance via autocrine and paracrine stimulation of cancer cells and pancreatic stellate cells.

## RESULTS

### Up-regulation of histone modifying enzymes in GEM-resistant pancreatic cancer cells

**Figure 1:**
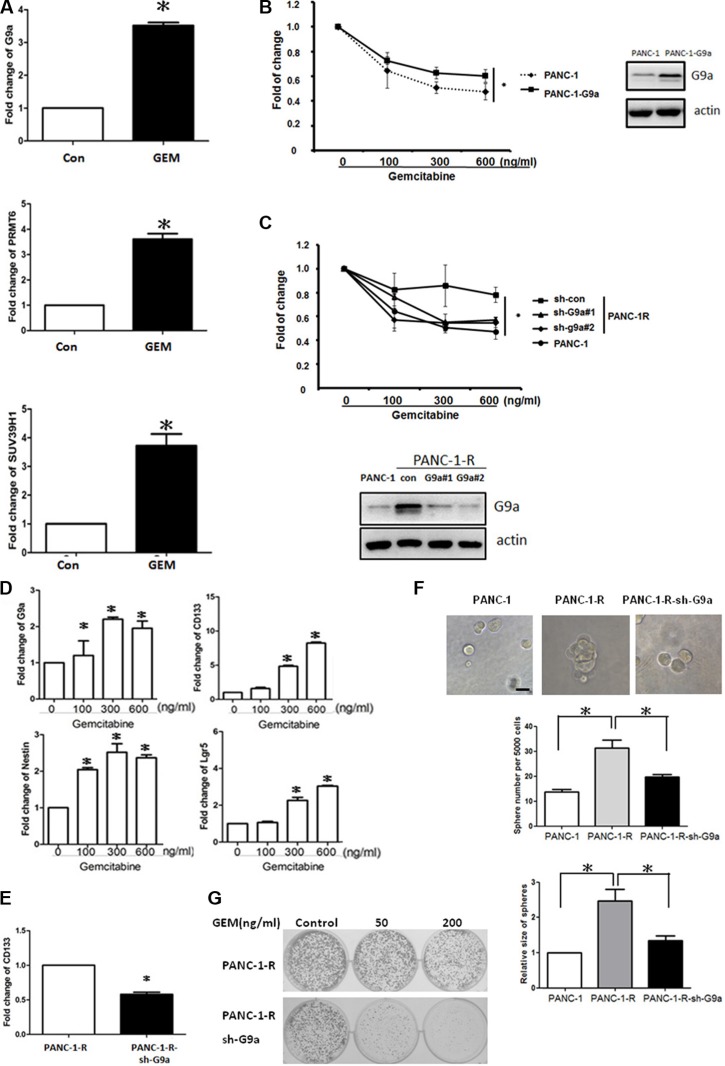
G9a increased GEM resistance and stem-like properties in pancreatic cancer cells (**A**) Expression of *G9a*, *PRMT6* and *SUV39H1* in parental PANC-1 (Con) and GEM-resistant PANC-1-R cells (GEM) were determined by RT-qPCR analysis. Columns represented the mean of triplicate PCR assays and normalized to GAPDH. *P < 0.05. (**B**) PANC-1 and G9a-overexpressing PANC-1 cells were treated with different concentrations of GEM for 48 h and cell viability was determined by MTT assay. *P < 0.05. (**C**) PANC-1-R cells were infected with control shRNA (sh-con) or various G9a shRNAs (sh-G9a#1 and sh-G9a#2) for 48 h and treated with different concentrations of GEM for another 48 h. Cell viability was determined by MTT assay. *P < 0.05. The protein level of G9a was examined by Western blot analysis (low panel). (**D**) PANC-1 cells were continuously incubated with the indicated concentrations of GEM for 10 days. Expression of *G9a, CD133, nestin* and *Lgr5* were determined by RT-qPCR. Columns represented the mean of triplicate PCR assays and normalized to GAPDH. *P < 0.05. (**E**) Expression of *CD133* mRNA in PANC-1-R and G9a-depleted PANC-1-R cells was determined by RT-qPCR analysis. *P < 0.05. (**F**) Cells were cultured in low attachment plates and number and size of the spheres were analyzed after 14 days. Results from three independent assays were expressed as Mean ± SE. *P < 0.05. (**G**) 1 × 10^3^ cells of PANC-1-R and PANC-1-R-sh-G9a cells were seed into 6 cm dish and continuous incubated with the indicated concentrations of GEM for 2 weeks to study the clonogenic activity.

We had previously established a GEM-resistant cell line (PANC-1-R) from the parental human PANC-1 pancreatic cancer cells [21]. To identify novel proteins involving in GEM resistance, we investigated the changes of histone modifying enzymes by using PCR array. As shown in Table 1, we found that 21 histone modifying enzymes including demethylases, methyltransferases, acetyltransferases, kinases and ubiquitin ligases were increased (> 2-fold) in GEM-resistant PANC-1-R cells. We performed quantitative RT-PCR to confirm the results of PCR array and found a similar increase of G9a, PRMT6 and SUV39H1 (Figure 1A). Among these molecules, we first focused on the H3K9 methyltransferase G9a because this enzyme has been shown to play an oncogenic role in several cancers and is a potential target for drug development [22].

**Table 1:** Alterations of chromatin-modifying enzymes in Gemcitabine-resistant PANC-1-R cells

Gene	Fold change	Protein function
PRMT3	3.49 ± 1.76	Histone Methyltransferases
PRMT6	3.38 ± 0.86	
SETD7	2.84 ± 1.23	
DOT1L	2.63 ± 0.45	
G9a	2.29 ± 0.3	
SMYD3	2.29 ± 0.26	
SETD2	2.26 ± 0.06	
SETD5	2.11 ± 0.59	
SETD8	2.33 ± 0.53	
SUV39H1	2.00 ± 0.12	
KDM5C	4.49 ± 0.72	DNA/Histone Demethylases
DZIP3	2.55 ± 0.12	Histone Ubiquitination
RNF20	2.14 ± 0.25	
NEK6	2.54 ± 0.055	Histone Phosphorylation
AURKC	2.13 ± 0.58	
RPS6KA3	2.02 ± 0.17	
NCOA3	2.37 ± 0.79	Histone Acetyltransferases
KAT2B	2.88 ± 1.62	
KAT6B	2.18 ± 0.76	
HDAC2	2.34 ± 0.61	Histone Deacetylases
HDAC11	2.28 ± 0.81	

We investigated whether overexpression of G9a increased cell survival under GEM treatment. As shown in Figure 1B, cells stably expressing G9a increased the resistance to GEM. Conversely, knockdown of G9a enhanced the sensitivity of PANC-1-R cells to GEM (Figure 1C). These data suggested that G9a may be involved in the regulation of GEM resistance.

### G9a was upregulated by GEM challenge and enhanced cancer stemness

Cancer cells with stemness properties have been shown to display high resistance to chemotherapeutic agents. PANC-1 cells were continuously incubated with different concentrations of GEM for 10 days and the surviving cells were harvested for the analysis of G9a and stemness genes. As shown in Figure 1D, G9a was significantly up-regulated in the surviving cells. In addition, the expression of three stemness markers of pancreatic cancer including CD133, nestin and Lgr5 was also up-regulated suggesting GEM treatment may stimulate the stem-like properties of cancer cells and enrich a population of cancer stem cells (CSCs) with high drug resistance. On the contrary, depletion of G9a reduced the expression of CD133 in PANC-1-R cells (Figure 1E). Moreover, the sphere number and size formed by PANC-1-R cells was about 2.5-fold higher than that of PANC-1 cells and knockdown of G9a in PANC-1-R cells significantly reduced the sphere forming activity (Figure 1F). Clonogenic assay also showed that G9a depletion sensitized PANC-1-R cells to GEM (Figure 1G).

We also validated the role of G9a in cancer stemness by studying another GEM-resistant human pancreatic cancer cell line (Mia-paca-2-R) derived from the parental Mia-paca-2 cells. Compared to the parental cells, the expression of G9a was upregulated by 3.5-fold in Mia-paca-2-R cells (Supplementary Figure S1A). A G9a specific inhibitor UNC0638 also decreased the proliferation of Mia-paca-2-R cells in a dose-dependent manner and sensitized the cells to GEM treatment (Supplementary Figure S1B). In addition, UNC0638 reduced the sphere forming activity of Mia-paca-2-R cells and co-treatment of UNC0638 and GEM suppressed the sphere number by 75–80% when compared to the control group (Supplementary Figure S1C).

### IL-8 is a mediator of G9a-induced GEM resistance

**Figure 2:**
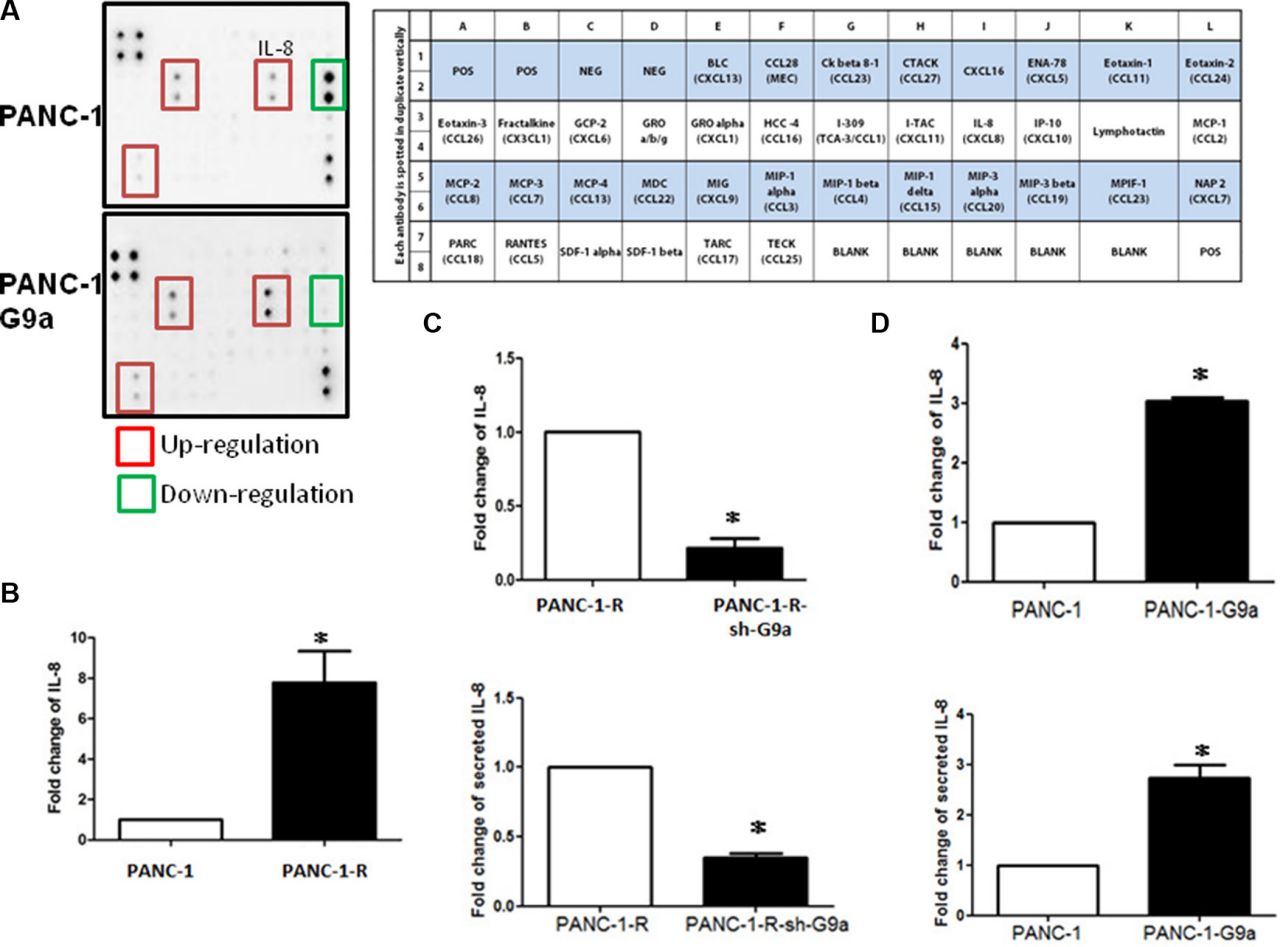
IL-8 is a target gene regulated by G9a in pancreatic cancer cells (**A**) The conditioned media of PANC-1 and G9a-overexpressing PANC-1 cells were collected and the secreted cytokines were determined by using human cytokine array. Up-regulated or down-regulated cytokines were labeled by red and green square mark. The cytokines tested in the array were shown in the right panel. (**B**) The *IL-8* mRNA level of PANC-1 and PANC-1-R cells was compared by using RT-qPCR assay. Columns represented the mean of triplicate PCR assays and normalized to GAPDH. *P < 0.05. (**C**) The *IL-8* mRNA level in cells and the secreted IL-8 protein in the conditioned media were studied by RT-qPCR assay (top panel) and by ELISA assay (bottom panel). The IL-8 level of PANC-1-R cells was defined as 1, and relative level of G9a-depleted PANC-1-R cells was shown. *P < 0.05. (**D**) The *IL-8* mRNA level in cells and the secreted IL-8 protein in the conditioned media of PANC-1 and G9a-overexpressing PANC-1 cells were studied by RT-qPCR assay (top panel) and by ELISA assay (bottom panel). Results from three independent assays were expressed as Mean ± SE. *P < 0.05.

**Figure 3:**
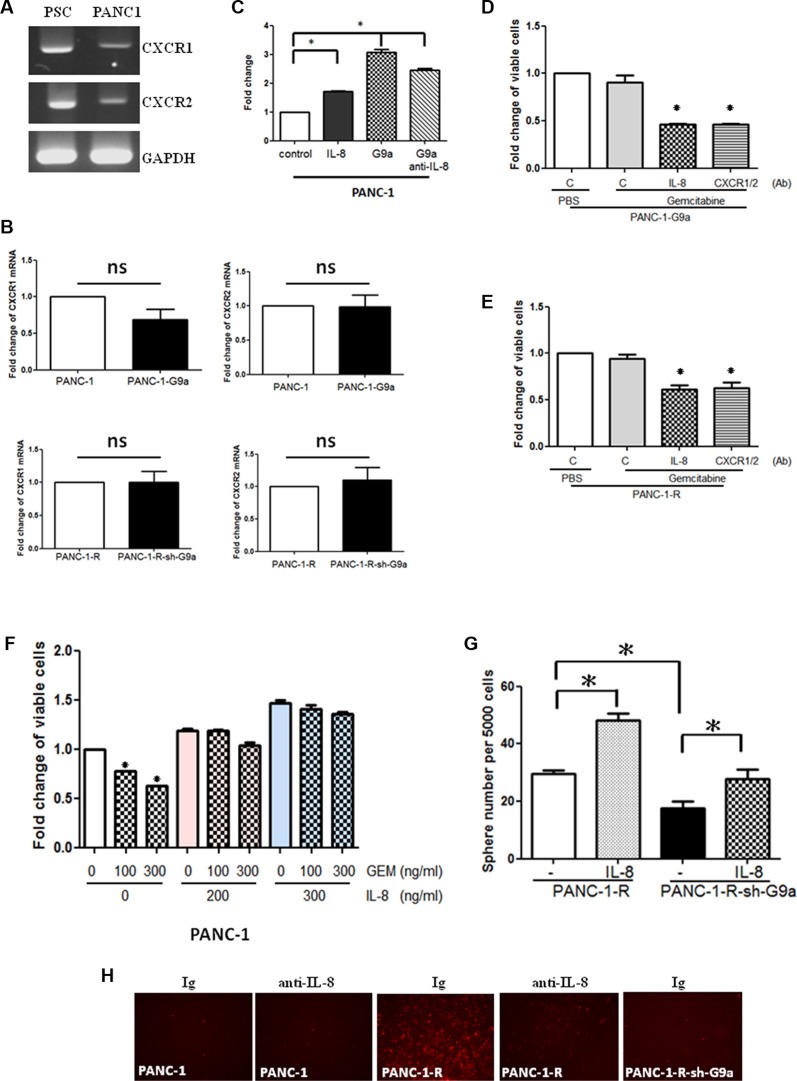
G9a-induced IL-8 increased GEM resistance and trans-endothelial invasion (**A**) Expression of *CXCR1* and *CXCR2* mRNA in PANC-1 cells and pancreatic satellite cells (PSC) was determined by RT-PCR analysis. (**B**) The expression of CXCR1 and CXCR2 in PANC-1, PANC-1-G9a, PANC-1-R and PANC-1-R-shG9a cells was studied by RT-qPCR analysis. Results from three independent assays were expressed as Mean ± SE. ns: not significant. (**C**) PANC-1 or G9a-overexpressing PANC-1 cells were treated with IL-8 (200 ng/ml) or anti-IL-8 (0.5 mg/ml) for 48 h and cellular proliferation was studied by MTT assay. *P < 0.05. (**D**) G9a-overexpressing PANC-1 cells were treated with GEM in combination with nonimmune immunoglobulin (C), anti-IL-8, or anti-CXCR1+anti-CXCR2 antibodies. Cell viability was assessed after treatment for 48 h by MTT assay. *P < 0.05. (**E**) PANC-1-R cells were treated with GEM in combination with nonimmune immunoglobulin (C), anti-IL-8, or anti-CXCR1+anti-CXCR2 antibodies. Cell viability was assessed after treatment for 48 h by MTT assay. *P < 0.05. (**F**) PANC-1 cells were pre-treated without or with different concentrations of IL-8 for 24 h and then incubated with 100 or 300 ng/ml of GEM for another 48 h. Cell viability was studied by MTT assay. *P < 0.05. (**G**) PANC-1-R and PANC-1-R-shG9a cells were cultured in low attachment plates in the presence or absence of IL-8 (200 ng/ml) for 14 days. The number of the spheres was counted. *P < 0.05. (**H**) PANC-1, PANC-1-R and PANC-1-R-shG9a cells were pre-incubated with nonimmune immunoglobulin (Ig) or anti-IL-8 antibody and subjected to trans-endothelial migration assay as described in MATERIALS and METHODS.

To identify the molecules that mediated G9a-induced chemoresistance, we first focused on soluble factors including growth factors and cytokines. Our array-based approach demonstrated that three cytokines including IL-8 were upregulated in PANC-1-G9a cells (Figure 2A). We checked the levels of IL-8 in PANC-1 and PANC-1-R cells and found an 8-fold increase of IL-8 mRNA in PANC-1-R cells (Figure 2B). A three-fold increase of IL-8 was found in Mia-paca-2-R cells (Supplementary Figure S2A). Knockdown of G9a significantly reduced IL-8 mRNA level in PANC-1-R cells and the secreted IL-8 in the conditioned medium (Figure 2C). Conversely, ectopic expression of G9a in PANC-1 cells upregulated IL-8 expression and increased secreted IL-8 in the culture medium (Figure 2D). Interestingly, we found that both pancreatic cancer cells and PSCs expressed IL-8 receptors CXCR1 and CXCR2 implicating the possibility that G9a-increased IL-8 could affect cancer cells and PSCs simultaneously (Figure 3A). Ectopic expression of G9a in PANC-1 or knockdown of G9a in PANC-1-R cells did not change the CXCR1 and CXCR2 expression (Figure 3B). Addition of IL-8 recombinant protein stimulated the proliferation of PANC-1 cells (Figure 3C). Overexpression of G9a increased the growth of PANC-1 cells which could be attenuated by anti-IL-8 antibody indicating IL-8 is a downstream effector for G9a to promote cellular proliferation (Figure 3C). In addition, increase of GEM resistance in PANC-1 cells by overexpression of G9a was reduced by IL-8 antibody (Figure 3D). Co-treatment of CXCR1 and CXCR2 antibodies also decreased the G9a-induced GEM resistance to a similar magnitude. Similarly, neutralization of IL-8 produced by PANC-1-R cells also increased the cytotoxic effect of GEM (Figure 3E). These data suggested that IL-8 increased by G9a overexpression could enhance GEM resistance in cancer cells via autocrine IL-8/CXCR1/2 stimulation. However, it should be noted that IL-8 or CXCR1/2 antibodies only partially reversed the resistance suggesting the involvement of other molecules in G9a-induced GEM resistance.

To further confirm the effect of IL-8 on GEM resistance, we pre-treated PANC-1 cells with IL-8 for 24 h and then incubated with different does of GEM for another 48 h. As shown in Figure 3F, pretreatment of IL-8 partly protected GEM-induced cell death. We also found that IL-8 recombinant protein could rescue the reduction of sphere formation by G9a knockdown suggesting IL-8 acts downstream of G9a (Figure 3G). Both IL-8 antibody and CXCR1/CXCR2 antibody combination significantly reduced the sphere number and size of G9a-overexpressing cells indicating that IL-8 is a mediator for G9a to promote cancer stemness via an IL-8/CXCR1/2 signaling axis (Supplementary Figure S3). Similarly, UNC0638 and IL-8 antibody also significantly decreased the sphere forming activity of Mia-paca-2-R cells (Supplementary Figure S2B).

Interesting, our data also showed that PANC-1-R cells exhibited higher trans-endothelial migration ability than PANC-1 cells (panel 1 and 3, Figure 3H). Addition of IL-8 neutralizing antibody significantly reduced the invasion of PANC-1-R cells (panel 3 and 4, Figure 3H). Notably, the depletion of G9a also dramatically suppressed the trans-endothelial migration ability (panel 3 and 5, Figure 3H). These data suggested IL-8 is involved in G9a-induced proliferation, trans-endothelial invasion, and GEM resistance of pancreatic cancer cells.

### IL-8 induces PSC activation and extracellular matrix deposition

**Figure 4:**
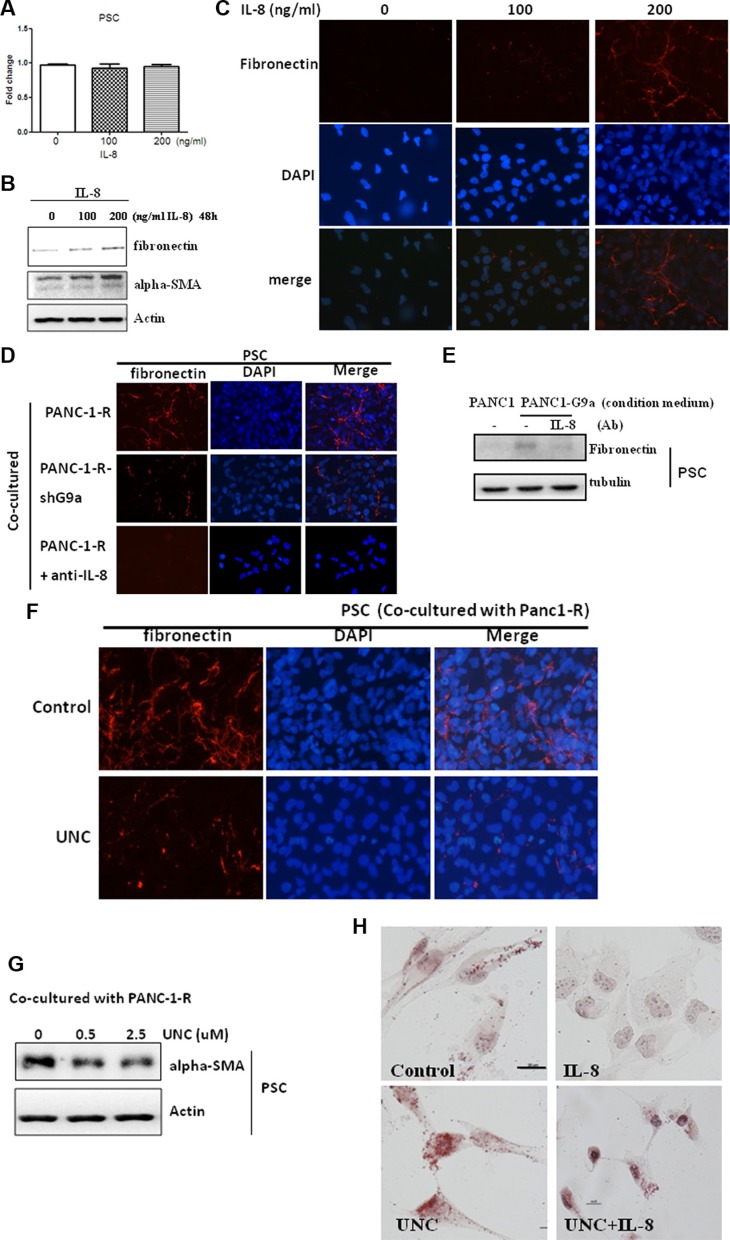
G9a-induced IL-8 promoted PSC activation (**A**) PSC cells were treated with indicated concentrations of recombinant IL-8 for 48 h and cellular proliferation was assessed. (**B**) PSC cells were treated with indicated concentrations of IL-8 for 48 h, and the protein level of fibronectin and α-SMA were studied by Western blot analysis. (**C**) PSC cells were treated with indicated concentration of IL-8 for 48 h and the expression and deposition of fibronectin in PSC cells was analyzed by immunofluorescence. (**D**) PANC-1-R and G9a-depleted PANC-1-R cells were seeded into upper inserts of transwell plates for 24 h and co-cultured with PSC cells for another 48 h. The expression and deposition of fibronectin in PSC cells was analyzed by immunofluorescence. (**E**) The conditioned medium collected from PANC-1 or G9a-overexpressing PANC-1 cells were pre-incubated with non-immune IgG (—) or IL-8 antibody for 4 h and were used to treat PSC cells. After 48 h, the protein level of fibronectin was investigated by Western blot analysis. (**F**) PANC-1-R cells were treated without or with UNC0638 (2.5 μM) for 24 h and co-cultured with PSC cells for another 48 h. The expression and deposition of fibronectin in PSC cells was analyzed by immunofluorescence. (**G**) PANC-1-R cells were treated with indicated concentration of UNC for 24 h and co-cultured with PSC for another 48 h. The expression of α-SMA in PSC cells were analyzed by Western blot. (**H**) PSC cells were treated with UNC0638 (2.5 μM) or IL-8 (200 ng/ml) for 48 h and the number of lipid droplets was stained by Oil Red dye. Scale bar, 5 micrometer.

Pancreatic cancer presents a dense desmoplastic stroma consisting abundant extracellular matrix proteins released by activated PSC to reduce the penetration of chemotherapeutic agents which leads to increased drug resistance. Because we found PSC expressed high level of CXCR1 and CXCR2 (Figure 3A), the effect of IL-8 on PSC activation was investigated. As shown in Figure 4A, IL-8 did not stimulate PSC proliferation. However, IL-8 induced PSC activation in a dose-dependent manner as evidenced by the up-regulation of alpha-smooth muscle actin (α-SMA) and fibronectin (Figure 4B). The deposition of fibronectin surrounding the PSCs was also increased by IL-8 (Figure 4C). Co-culture of IL-8-overexpressing PANC-1-R cells with PSC induced abundant fibronectin deposition while co-culture with G9a-depleted PANC-1-R cells showed little effect (Figure 4D). Neutralization of IL-8 in the conditioned medium of PANC-1-R cells decreased fibronectin deposition (Figure 4D) and also reduced the stimulation of fibronectin expression by the conditioned medium in PSC cells (Figure 4E).

UNC0638 also significantly inhibited fibronectin deposition and PSC activation induced by co-culture (Figure 4F and 4G). Finally, we studied lipid droplet decomposition to confirm the activation of PSC by IL-8 and found that IL-8 reduced the number of lipid droplet stained by Oil Red which could be fully reversed by UNC0638 (Figure 4H). Collectively, these data suggested that IL-8 released by pancreatic cancer cells stimulates PSC activation via paracrine mechanism and inhibition of G9a in cancer cells reduces IL-8 secretion, PSC activation, and matrix protein deposition.

### Inhibition of G9a overcomes GEM resistance in orthotopic animal study

**Figure 5:**
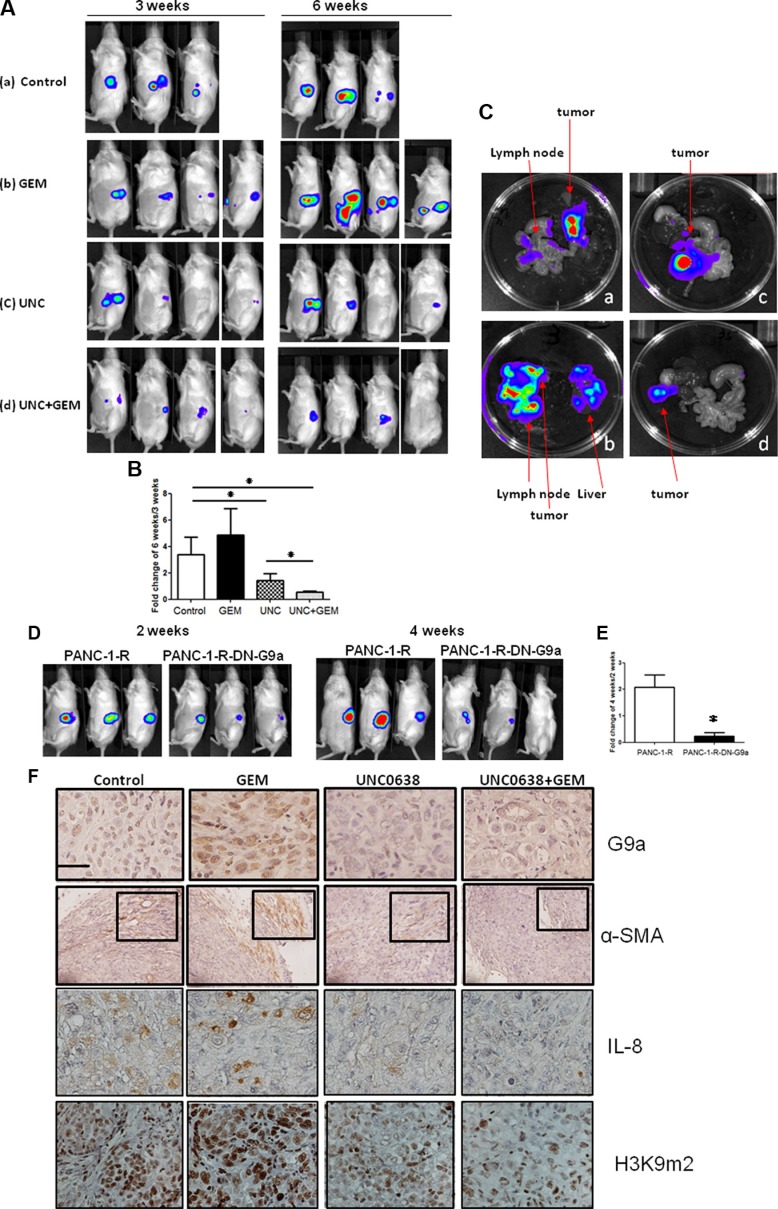
G9a inhibitor overrided GEM resistance and suppressed tumor growth and metastasis of GEM-resistant pancreatic cancer cells (**A**) Tumor growth was examined by biofluorescence imaging. Fluorescent images of the whole body of tumor-bearing mice received different treatments as indicated in MATERIALS and METHODS at 3 and 6 weeks after orthotopic inoculation of PANC-1-R cancer cells into the pancreas. (**B**) The photon number fluxed from the tumors in the control or drug-treated animals were measured at week 3 and 6. The increase of photon number (6 weeks versus 3 weeks) was compared in different experimental groups. The statistical difference between different groups was assessed. Control group: *n*= 3 and other drug-treated groups: *n* = 4. *P < 0.05. (**C**) Organs of tumor-bearing mice were shown to demonstrate tumor metastasis. GEM treatment alone induced stronger local invasion and distant metastasis to liver. Combination of GEM and UNC significantly reduced tumor growth and metastasis. a:control group; b:GEM treatment group; c:UNC0638 treatment group and d:GEM+UNC group. (**D**) Fluorescent images to demonstrate the tumor growth induced by PANC-1-R cells or PANC-1-R cells overexpressing dominant-negative G9a (PANC-1-R-DN-G9a) in mice. (**E**) The photon number fluxed from the tumors in the animals injected with PANC-1-R or PANC-1-R-DN-G9a cells were measured at week 2 and 4. The increase of photon number (4 weeks versus 2 weeks) was compared in two experimental groups. The statistical difference between two groups was assessed. PANC-1-R group: *n* = 3; PANC-1-R-DN-G9a group: *n* = 3. *P < 0.05. (**F**) Representative images of immunohistochemical staining for G9a, α-SMA, IL-8 and H3K9 di-methylation (H3K9me2) in the tumors of different treatment groups. Scale bar, 20 micrometer. The staining of H3K9me2 is monitored to validate the inhibitory effect of UNC0638 on G9a enzymatic activity.

To validate G9a is a therapeutic target to override GEM resistance as demonstrated in our cell-based assays, we performed orthotopic animal study to determine whether G9a inhibitor could cooperate with GEM to inhibit growth and metastasis of drug-resistance cells. PANC-1-R cells were injected into the pancreas of mice. One week later, mice were randomly distributed into four groups to receive DMSO (control), GEM, UNC0638 and GEM+UNC0638 treatment. As shown in Figure 5A, GEM treatment did not significantly inhibit PANC-1-R tumors. UNC0638 alone showed partial inhibition and GEM+UNC0638 demonstrated a significant suppression of tumor growth. At 6 weeks, tumors of the control group grew continuously (Figure 5A). Unexpectedly, GEM treatment boosted the growth of PANC-1-R tumors. UNC0638 induced shrinkage of tumors in 75% (3/4) of the animals and combination of GEM and UNC0638 potently inhibited tumor growth. We also quantified the photon number fluxed from the tumors and the data of the animals measured at week 3 and 6 were compared. As shown in Figure 5B, a 3.5-fold increase of photon counts was detected in the control growth consisting with the upregulation of image intensity in Figure 5A. GEM treatment induced a higher (about 5-fold) increase of photon counts in animals. UNC0638 alone suppressed the increase to 1.7-fold and UNC0638+GEM reduced the increase to only 20%.

We checked regional metastasis of tumors in the animals and found that tumors of the control group were mainly located within the pancreas with some local invasions as the IVIS image showed weak signals in the regional lymph nodes (group a, Figure 5C). GEM treatment alone did not exhibit any suppressive effect and eventually promoted local invasion and metastasis to liver (group b, Figure 5C). Tumors of the UNC0638 group were restricted in the pancreas of the animals (group c, Figure 5C). Tumor size was dramatically reduced in the GEM+UNC0638 group and no local invasion was found in the animals (group d, Figure 5C). Consistent to these results, ectopic expression of dominant-negative G9a (without enzymatic activity) in PANC-1-R cells also reduced their tumor-forming activity in animals (Figure 5D). Compared the photon counts of the animals at 2 and 4 weeks after cancer cell injection, a 2-fold increase was found in the control group while only a 15% increase was detected in the dominant-negative G9a group (Figure 5E).

Immunohistochemical analyses revealed the positive staining of G9a and IL-8 in the control and GEM-treated groups and the level of these two proteins was reduced in the UNC0638 and GEM+UNC0638 groups (Figure 5F). It should be noted that the levels of G9a and IL-8 were always much higher in the GEM-treated group compared to the control group. These data implied that continuous presence of GEM is required for the maintenance of drug-resistant phenotype and the resistance may be decreased after the removal of GEM for a period. Similar results were also found in the cultured cells. We found the expression of α-SMA in the stellate cells of the control and GEM-treated tumors suggesting the activation of PSC. Conversely, very low or negative α-SMA signal was observed in the UNC0638 and GEM+UNC0638 groups. To validate the inhibitory effect of UNC0638 on G9a activity, we examined the H3K9 di-methylation status. As shown in Figure 5F, the control and GEM-treated groups showed strong H3K9 di-methylation staining while the UNC0638 and the GEM+UNC0638 groups demonstrated weak staining. The signal intensity of G9a in the tumors matched the signal intensity of H3K9 dimethylation confirming the inhibition of G9a activity by UNC0638. These data suggested that combination of GEM and G9a inhibitor could suppress tumor growth and metastasis of GEM-resistant cancer cells.

### Increase of G9a is correlated with poor clinical outcome in pancreatic cancer

**Figure 6:**
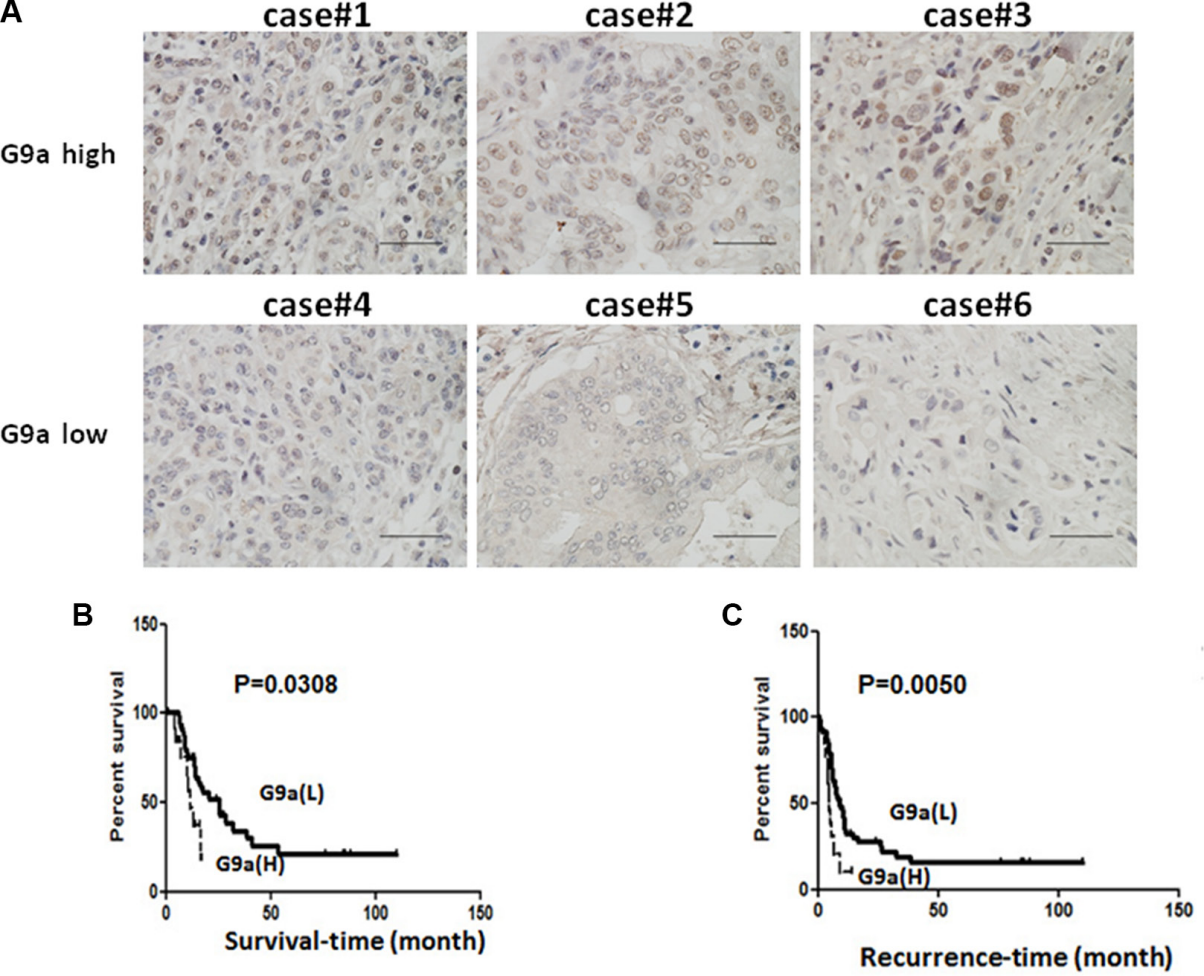
High levels of G9a expression was correlated with poor clinical outcome in pancreatic cancer patients (**A**) Representative images of pancreatic tumor tissues with high or low expression of G9a. (**B**) Kaplan-Meier analysis to show the overall survival of pancreatic cancer patients with high or low expression of G9a. (**C**) Recurrent time of pancreatic cancer patients with high or low expression of G9a. Scale bar, 20 micrometer.

To validate the clinical significance of G9a, we examined the correlation between G9a expression and clinical characteristics. Sixty pancreatic cancer patients were included and their clinical information was shown in Table 2. Up-regulation of G9a was detected in 23.3% (14/60) of the tumor tissues and the representative pictures of high or low G9a staining were shown in Figure 6A. G9a expression was not associated with sex, age, tumor size, pathological grade, lymph node metastasis, and tumor stage (Table 3). The lack of correlation between G9a and tumor size or lymph node metastasis could be due to the small case number investigated in this study. However, Kaplan-Meier plot demonstrated that high G9a expression was associated with a shorter survival time and a quick recurrence (Figure 6B and 6C).

**Table 2:** Clinical information of pancreatic cancer patients

Characteristic	No. of patients (*N* = 60)	Percentage (%)
**Sex**		
Men	37	61.7
Women	23	38.3
**Age**		
Mean ± SD, year	62.9 ± 12	
**Tumor size**		
≤ 3 cm	23	38.3
> 3 cm	37	61.7
**Pathological grade**		
Well, moderate	53	88.3
Poor	7	11.7
**Depth of invasion**		
T1, T2	9	15
T3, T4	51	85
**Lymph nodemetastasis**		
Negative	28	46.7
Positive	32	53.3
**Stage**		
I, II	57	95
III, IV	3	5

**Table 3:** Association of G9a expression with clinicopathological parameters in pancreatic cancer patients

	G9a		*P*
	Low	High	
**Sex**			
Men	29	8	0.6909
Women	17	6	
**Age**			
< 60 years old	21	4	0.2563
≥ 60 years old	25	10	
**Tumor size**			
≤ 3 cm	18	5	0.8109
>3 cm	28	9	
**Pathological grade**			
Well, moderate	41	12	0.7273
Poor	5	2	
**Depth of invasion**			
T1, T2	6	3	0.7180
T3, T4	37	14	
**Lymph node metastasis**			
Negative	22	6	0.7441
Positive	24	8	
**Stage**			
I, II	44	13	0.6743
III, IV	2	1	

## DISCUSSION

In the present study, we profiled the expression of histone modifying enzymes by using array-based approach to elucidate the alterations of these enzymes in GEM-resistant pancreatic cancer cells. To the best of our knowledge, this is the first study to link histone modifying enzymes with GEM-resistance in a global way. Among the altered enzymes, several potential therapeutic targets warrant further investigation. For example, SUV39H1 is a methyltransferase responsive for the introduction of trimethylation of lysine-9 of histone H3 (H3K9) [23]. Recently, a specific SUV39H1 inhibitor chaetocin has been reported [24, 25]. Our previous study found that chaetocin induced differentiation of acute myeloid leukemia cells and acted synergistically with other epigenetic drugs to kill leukemia cells [26]. Another example is DOT1L, a H3K79 methyltransferase and a core component of the mixed lineage leukemia (MLL) protein complex, which involved in the transforming activity of various MLL-rearranged fusion oncogenes [27]. The development of DOT1L inhibitors is a hot area in anti-cancer drug research. Currently, clinical trial has been initiated with a potent DOT1L inhibitor EPZ-5676 for the treatment of hematological malignancies [28]. We picked up G9a as a candidate of high priority because this enzyme is known to function as an oncogene in several cancers and potent inhibitors against this methyltransferase have been developed [29]. The role of other histone modifying enzymes in the tumorigenesis and drug resistance of pancreatic cancer is under investigation in our laboratory.

Previous studies have shown that G9a perpetuates malignant phenotypes through multiple mechanisms. G9a depletion restored a cohort of tumor suppressor genes including E-cadherin, DUSP5, SPRY4, and PPP1R15A in ovarian cancer [30]. G9a also inhibited the expression of cell adhesion molecule EP-CAM in lung cancer to increase aggressiveness [31]. In addition, G9a regulated the expression of FAS to modulate the resistance of metastatic colon cancer cells to 5-fluorouracil *in vitro* and *in vivo* [32]. The regulation of cancer stemness by G9a was first reported in basal-like breast cancer cells [33]. The study showed that a repression complex Snail-G9a- DNA methyltransferase 1 may repress fructose-1,6-biphosphatase expression to change cellular metabolism and to increase cancer stem cell characters. Two recent studies demonstrated that G9a plays an important role in the maintenance of cancer stem cell-like properties in head and neck cancer and in obesity-mediated breast cancer progression [34, 35]. Conversely, G9a was shown to inhibit the expression of CD133 and SOX2 to reduce the self-renewal of glioma cancer stem cells [36]. We provide evidence that G9a expression correlated with the expression of the stemness genes including CD133, nestin and Lrg5 and inhibition of G9a in pancreatic cancer cells attenuated CD133 expression suggesting a role of G9a in the regulation of stemness in this cancer.

Although G9a shows oncogenic activity in cancers, its role in drug resistance is largely unknown. Candilaria et al demonstrated that hydralazine, a smooth muscle relaxant and vasodilator, could reverse GEM resistance in cervical cancer cells [37]. They found hydralazine could inhibit G9a activity *in vitro* and in cells and suggested that G9a may be a target for hydralazine to counteract GEM resistance. However, hydralazine is not a specific G9a inhibitor and could target a number of intracellular molecules to elicit its biological effect. Therefore, the contribution of G9a in GEM resistance is still unclear. By using a specific inhibitor (UNC0638) and different approaches (shRNA knockdown, overexpression and animal study), we demonstrated for the first time that G9a is an important determinant of GEM resistance.

GEM resistance *in vivo* may generate from (1) genetic alterations in cancer cells to modulate drug transports and DNA repair enzymes, (2) change of stromal components to interfere drug penetration, and (3) crosstalk between cancer and stromal cells to against drug cytotoxicity via exchange of survival factors or direct cell-cell contact. We focused on the cancer/stroma crosstalk and identified IL-8 as a downstream effector of G9a to increase GEM resistance by both autocrine and paracrine stimulation. The IL-8/CXCR1/CXCR2 signaling axis is critical for the establishment of stem-like properties in breast cancer [38]. How IL-8 is upregulated in cancer cells is under intensive investigation. Hypoxia and acidosis have been shown to increase IL-8 expression and contribute to aggressive behaviors of pancreatic cancer cells [39, 40]. We provided evidence that overexpression of G9a induced IL-8 expression in pancreatic cancer cells through transcriptional activation. We further showed that G9a inhibition decreased IL-8 expression and enhanced the sensitivity of drug-resistant cancer cells to GEM. Moreover, depletion of secreted IL-8 by neutralizing antibody also reduced the resistance to GEM suggesting cancer cells may enhance GEM resistance via an autocrine IL-8/CXCR1/2 loop. Importantly, we also found that IL-8 released by cancer cells induced activation of PSC via a paracrine mechanism to produce abundant extracellular matrix proteins that may interfere drug penetration into tumors. We hypothesize that GEM treatment stimulates G9a expression in pancreatic cancer cells that in turn enriches a population of G9a-overexpressing stem-like cancer cells to produce IL-8 to modulate tumor microenvironment to increase GEM resistance. This hypothesis is evidenced by the animal study that G9a inhibitor in combination with GEM reduces IL-8 production and decreases tumor growth, lymph node invasion and distant metastasis of GEM-resistant cancer cells.

It is obvious that IL-8 is not the only effector controlled by G9a to promote GEM resistance. In our cytokine arrays, several potential molecules like CXCL5 and GRO (also known as CXCL1) may also involve in G9a-induced GEM resistance. In addition to soluble factors, the importance of cellular proteins that promote cell-cell contact could not be underscored. Further studies will be needed to clarify the role of these molecules *in vitro* and *in vivo*. Collectively, our results provide a molecular basis by which G9a promotes the resistance to chemotherapeutic drugs by changing cancer cells and stromal cells simultaneously. More importantly, we identify G9a inhibitors as potential compounds to override GEM resistance in pancreatic cancer therapy.

## MATERIALS AND METHODS

### Cell lines, reagents and plasmids

Pancreatic cancer cell lines were grown in Dulbecco's modified Eagles medium containing 10% fetal bovine serum. A gemcitabine-resistant cell line (PANC-1-R) was established from the parental human PANC-1 pancreatic cancer cells [21]. Pancreatic satellite cells (PSC) were kindly provided from Kelvin K. Tsai (National Health Research Institutes, Taiwan). RNA interference expression plasmid specific for G9a was purchased by the National RNAi Core Facility (Academia Sinica, Taiwan). The sources of antibodies used were: anti-G9a and anti-IL-8 were from Epitommics Burlingame, CA, USA); Anti-α-SMA antibody was from Sigma. (St. Louis, MI, USA). UNC0638 was purchased from Cayman Company (An Arbor, MI, USA). GEM (Gemzar, Lilly) stocks (40 mg/ml) were stored in aliquots at 4°C until use. G9a shRNA plasmids were from National RNAi Core, Taiwan (G9a#1 sequence: GCTCCAGGAATTTAACAAGAT; G9a#2 sequence: CTCCAGGAATTTAACAAGATT).

### Lentivirus production and delivery shRNA

For lentivirus particle production, 293FT cells were plated in 10-cm dishes (1 × 10^6^ cells per dish) transfected by Lipofectamine 2000 (Invitrogen) according to the manufacturer's protocol. The following plasmids were used for transfection per 10-cm dish: 6 μg PLKO.1 puro-shG9a, 0.6 μg of the envelop plasmid pMD.G2 and 5.4 μg of the packaging vector pCMV delta8.9. The media were changed the next day, and virus were harvested by collecting the media at 48 and 72 h post-transfection, and then passed through 0.45-μm filter and stored at −80°C. For lentivirus transduction, PANC-1 cells were infected by media containing virus and polybrene (8 μg/ml). After 24 h, media were replaced with fresh medium containing 2.5 μg/ml of puromycin to select the successfully infected cells.

### PCR arrays and real-time RT-qPCR analysis

Total RNA was extracted using the RNeasy mini kit (Qiagen, Valencia, CA). Two hundred nanograms of total RNA from each sample were reverse-transcribed into cDNA using the RT2 first strand kit (Qiagen). The Human Epigenetic Chromatin Modification Enzymes RT^2^ Profiler™ PCR Array (Qiagen) was used to examine the mRNA levels of 84 key genes. Both β-actin and GAPDH were used as house-keeping genes. Several negative controls were included in each run. All PCR experiments were conducted with a StepOne Real Time PCR system (Applied Biosystems, Carlsbad, CA). The data analysis was performed using the ^ΔΔ^Ct based calculations. For real-time RT-qPCR analysis, total RNA was extracted and assays were done in triplicated by using the primers indicated as following. G9a-F: 5′-TGGGAAAGGTGACCTCAGAT-3′; G9a-R: 5′-TCCCTGACTCCTCATCTTCC-3′; Lgr5-F:5′-C CTTCCAACCTCAGCGTCTT-3′; Lgr5-R:5′-AGGGAT TGAAGGCTT CGCAA-3′; PRMT6-F:5′-CCTGGGTA TCCTTCGGAACT-3′; PRMT6-R:5′-CT CCTTCAGC CACTTGGTTC-3′;CD133-F:5′-GTCACCATTGACTTC TTGGTGCTGT-3′;CD133-R:5′-TGTCAGATGGAGTTA CGCAGGTTTC-3′;CXCR1-F:5′-CCAGTCCAGTTTGC TATGAGGT-3′;CXCR1-R:5′-TGTAGGAGGTAACACG ATGACG-3′;CXCR2-F:5′-CTTCTTCAGGGCACACTTC C-3′;CXCR2-R:5′-CAGAGCTCCAGCAAATGACA-3′; IL-8-F: 5′-CAGAGACAGCAGAGCACAC -3′; IL-8-R: 5′-AGTTCTTTAGCACTCCTTGGC-3′.

### Enzyme-linked immunosorbent assay (ELISA) for IL-8

PANC-1 cells stably expressing G9a expression vector or G9a shRNA plasmids were cultured in DMEM medium for 24 h. The conditioned medium was collected and centrifuged at 1500 rpm for 5 min to remove cell debris. IL-8 concentration was measured using Quantikines Human IL-8 Immunoassay kit (R&D, Minneapolis, MN, USA).

### Cell migration and invasion assay

Cell migration and invasion ability were performed as previously described [41]. Briefly, 5 × 10^3^ cells were seeded in 24-well transwell units with polycarbonate filters (pore size 8 μm) coated on the upper side with 1% gelatin (Sigma) for invasion assay for 24 h. After careful removal of the cells in the upper surface of the filters, migrated and invaded cells were fixed and stained with 0.05% Trypan Blue.

### Trans-endothelial migration assay

The *in vitro* trans-endothelial migration assay was followed using CytoSelect™ Tumor Transendothelial Migration Assay (Cell Biolabs, Inc., San Diego, CA). SVEC4-10 endothelial cells (5 × 10^4^ cells) were placed on top of a Matrigel-coated transwell (8 μm pore size) and allowed to adhere for 24 h. After that, PANC-1 or PANC-1-R cells (1 × 10^6^ cells/ml) were stained with CytoTracker and placed on top of upper chamber for another 24 h. Co-cultures were fixed with paraformaldehyde and analyzed by fluorescence microscope (Leica Microsystems, Richmond Hill, Ontario, Canada). In antibody inhibition studies, cancer cells were co-incubated with the control immunoglobulin (Ig) or anti-IL-8 blocking antibody (Genetex) before seeding.

### Cytokine antibody array

Proteins secreted from PANC-1 or G9a-overexpressing PANC-1 cells were compared by using the RayBio Human Chemokine Array C1 (RayBiotech Inc) according to the manufacture's instructions. In brief, cells were seeded into 10-cm dish. After 24 h, culture medium was changed to fresh medium with 1% FBS for another 24 h. The conditioned medium was then collected, concentrated and subjected to the antibody array incubation. Finally, the protein spots were detected using ECL Western blotting detection and quantified by a densitometer.

### Sphere formation assay

Cells were maintained as a monolayer in high glucose DMEM with 10% fetal bovine serum (FBS), 100 IU/ml penicillin G and 100 μg/ml streptomycin at 37°C in a humidified 5% CO2 incubator. Cells were collected and washed to remove serum, then suspended in serum-free DMEM/F12 supplemented with 100 IU/ml penicillin, 100 μg/ml streptomycin, 20 ng/ml human recombinant epidermal growth factor (hrEGF), 10 ng/ml human recombinant basic fibroblast growth factor (hrbFGF), 2% B27 supplement without vitamin A, 1% N2 supplement (Invitrogen, Carlsbad, CA, USA). Cells were subsequently cultured in ultra low attachment 6-well plates (Corning Inc., Corning, NY, USA) at a density (5,000 cells/well). After 14 days, number and size of the spheres were analyzed by *ImageJ* software.

### Orthotopic xenograft animal model

PANC-1-R cells were transduced with a retroviral vector encoding green fluorescence protein and firefly luciferase. Cells (1 × 10^6^ cells) in 35 μL Hank's balanced saline were inoculated into the pancreatic body of 8-week-old nonobese diabetic/severe combined immunodeficient mice. After one week, animals were randomly distributed into four groups to receive drugs via intraperitoneal injection: Group 1 served as the negative control and did not receive any treatment. Group 2 received gemcitabine (100 mg/kg per dose). Group 3 received UNC0638 (5 mg/kg per dose) and group 4 received GEM+UNC0638 co-treatment. Mice received intraperitoneal injection two times a week and tumor mass and distribution were assessed by bioluminescence (IVIS Imaging System, Caliper Life Sciences, Waltham, MA). After 6 weeks, mice were sacrificed for further analysis. All studies were approved by the Animal Care Committee of National Health Research Institutes.

### Patients and tissues

This study was approved by the Medical Ethics and the Human Clinical Trial Committee of National Cheng Kung University Hospital. Primary pancreatic adenocarcinoma tissues collected from sixty patients from National Cheng Kung University Hospital with clinicopathological information (including age, sex, tumor size, pathological grade, lymph node metastasis, and tumor staging as well as follow-up data were included in this study.

### Immunohistochemistry

Paraffin tumor samples were cut (4 μm in thickness) and deparaffined in xylene as described previously [42]. Endogenous peroxidase was blocked with 4% H_2_O_2_ for 15 min. Sections were stained with indicated primary antibodies, appropriate secondary antibodies and Envission system (Dako, Denmark). Finally, sections were counterstained with hematoxylin and analyzed by microscope.

### Statistics

The experiments were repeated at three times. Results are expressed as Mean + SE as indicated. Two-tailed Student's *t* test was performed to compare intergroup differences. The overall survival was analyzed by the Kaplan-Meier estimator and analyzed by the log-rank test. *P value* less than 0.05 was considered statistically significant.

## SUPPLEMENTARY MATERIALS FIGURES


