# Supplementary material for: The histone methyltransferase G9a as a therapeutic target to override gemcitabine resistance in pancreatic cancer
Source: Oncotarget. 2016 Aug 12;7(38):61136–51. doi: 10.18632/oncotarget.11256 (PMC5308641; doi:10.18632/oncotarget.11256)
Supplement: Supplementary file 1 [file oncotarget-07-61136-s001.pdf]

The histone methyltransferase G9a as a therapeutic target to override gemcitabine resistance in pancreatic cancer

Supplementary Materials

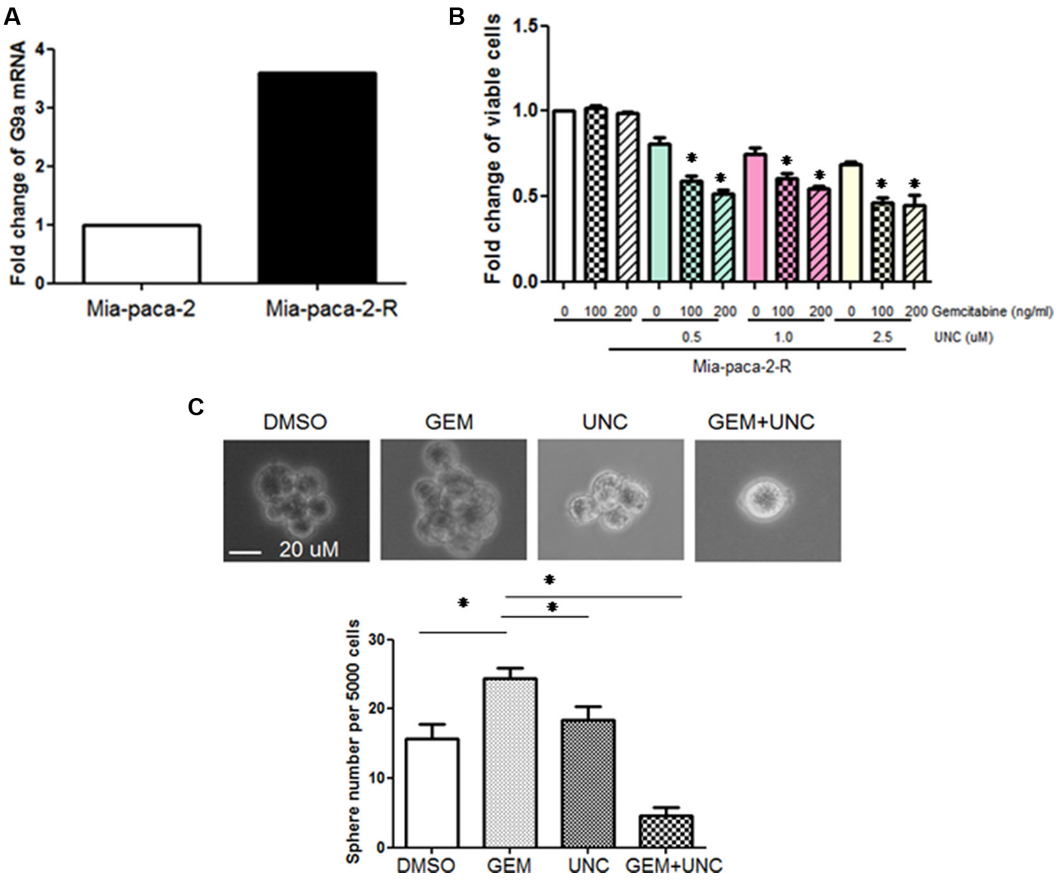

Supplementary Figure S1: Up-regulation of G9a in GEM-resistant Mia-paca-2-R pancreatic cancer cells and inhibition of viability and sphere formation of Mia-paca-2-R cells by G9a inhibitor UNC0638.

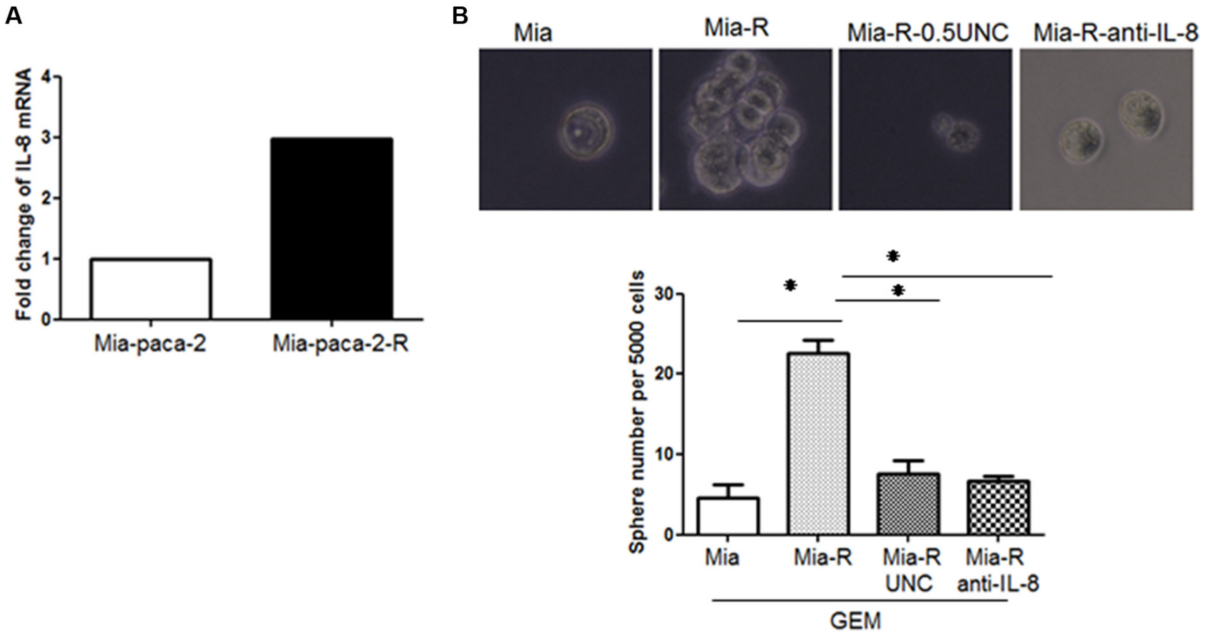

Supplementary Figure S2: Up-regulation of IL-8 in GEM-resistant Mia-paca-2-R pancreatic cancer cells and inhibition of sphere formation of Mia-paca-2-R cells by G9a inhibitor UNC0638 and anti-IL-8 antibody.

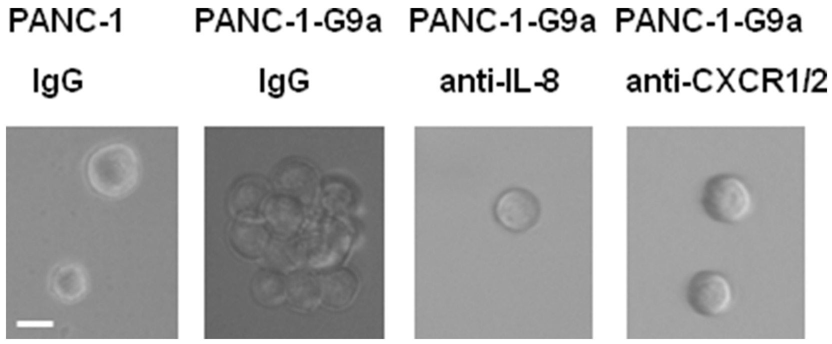

Supplementary Figure S3: Overexpression of G9a in PANC-1 cells increased sphere formation which could be inhibited by anti-IL-8 antibody or combination of anti-CXCR1 and anti-CXCR2 antibodies.
